# Prospective single-center study of health-related quality of life after COVID-19 in ICU and non-ICU patients

**DOI:** 10.1038/s41598-023-33783-y

**Published:** 2023-04-26

**Authors:** Johannes Herrmann, Kerstin Müller, Quirin Notz, Martha Hübsch, Kirsten Haas, Anna Schäfer, Julia Schmidt, Peter Heuschmann, Jens Maschmann, Matthias Frosch, Jürgen Deckert, Hermann Einsele, Georg Ertl, Stefan Frantz, Patrick Meybohm, Christopher Lotz

**Affiliations:** 1 Department of Anaesthesiology, Intensive Care, Emergency and Pain Medicine, University Hospital Würzburg, Julius-Maximilians-University Wuerzburg, Würzburg, Germany; 2 Institute for Clinical Epidemiology and Biometry, Julius-Maximilians-University Würzburg, Würzburg, Germany; 3 Clinical Trial Center, University Hospital Wuerzburg, Würzburg, Germany; 4 University Hospital Würzburg, Julius-Maximilians-University Würzburg, Würzburg, Germany; 5 University Hospital Würzburg, Julius-Maximilians-University Wuerzburg, Würzburg, Germany; 6 Department of Psychiatry, Psychosomatics and Psychotherapy, University Hospital Würzburg, Julius-Maximilians-University Würzburg, Würzburg, Germany; 7 Department of Internal Medicine II, University Hospital Würzburg, Julius-Maximilians-University Würzburg, Würzburg, Germany; 8 Comprehensive Heart Failure Center Würzburg (CHFC), University Hospital Würzburg, Julius-Maximilians-University Wuerzburg, Würzburg, Germany; 9 Department of Internal Medicine I, University Hospital Würzburg, Julius-Maximilians-University Würzburg, Würzburg, Germany; 10 Department of Anaesthesiology, Intensive Care, Emergency and Pain Medicine, University Hospital Würzburg, Oberduerrbacherstr. 6, 97080 Würzburg, Germany

**Keywords:** Health care, Public health, Quality of life

## Abstract

Long-term sequelae in hospitalized Coronavirus Disease 2019 (COVID-19) patients may result in limited quality of life. The current study aimed to determine health-related quality of life (HRQoL) after COVID-19 hospitalization in non-intensive care unit (ICU) and ICU patients. This is a single-center study at the University Hospital of Wuerzburg, Germany. Patients eligible were hospitalized with COVID-19 between March 2020 and December 2020. Patients were interviewed 3 and 12 months after hospital discharge. Questionnaires included the European Quality of Life 5 Dimensions 5 Level (EQ-5D-5L), patient health questionnaire-9 (PHQ-9), the generalized anxiety disorder 7 scale (GAD-7), FACIT fatigue scale, perceived stress scale (PSS-10) and posttraumatic symptom scale 10 (PTSS-10). 85 patients were included in the study. The EQ5D-5L-Index significantly differed between non-ICU (0.78 ± 0.33 and 0.84 ± 0.23) and ICU (0.71 ± 0.27; 0.74 ± 0.2) patients after 3- and 12-months. Of non-ICU 87% and 80% of ICU survivors lived at home without support after 12 months. One-third of ICU and half of the non-ICU patients returned to work. A higher percentage of ICU patients was limited in their activities of daily living compared to non-ICU patients. Depression and fatigue were present in one fifth of the ICU patients. Stress levels remained high with only 24% of non-ICU and 3% of ICU patients (p = 0.0186) having low perceived stress. Posttraumatic symptoms were present in 5% of non-ICU and 10% of ICU patients. HRQoL is limited in COVID-19 ICU patients 3- and 12-months post COVID-19 hospitalization, with significantly less improvement at 12-months compared to non-ICU patients. Mental disorders were common highlighting the complexity of post-COVID-19 symptoms as well as the necessity to educate patients and primary care providers about monitoring mental well-being post COVID-19.

## Introduction

Coronavirus disease 2019 (COVID-19) ranges from asymptomatic infection up to critical illness requiring hospitalization and intensive care unit (ICU) treatment. In a recent meta-analysis of 13,398 patients, the mortality rate of all COVID-19 patients admitted to the hospital was 11.5% and 40.5% among critically ill ICU patients^1^. However, COVID-19 does not end with survival to hospital discharge. Symptoms and organ dysfunction can persist as a post-COVID-19 syndrome. Problems include fatigue, muscle weakness, cognitive dysfunction, and posttraumatic stress disorder (PTSD)^2^. In a substantial part of the patients these long-term conditions may impact quality of life. Recent systematic reviews with a limited number of heterogenic studies and varying follow-ups up to 6 months concluded that post-COVID-19 health-related quality of life (HRQoL) remained poor^3,4^. In a multicenter study from six hospitals in China, HRQoL was still impaired in a 3-month follow-up. Most of these patients had mild to moderate COVID-19, whereas female gender and older age were risk factors for a reduced HRQoL^5^.

Quality of life itself is a multidimensional construct, which includes universal and emic properties. The World Health Organization definition of quality of life includes physical and mental health, social conditions, income, and environmental quality^6^. Nevertheless, in case of illness, almost all aspects can become health related. Moreover, physiologic measures often correlate poorly with functional capacity and well-being in daily activities^7^. As such, the evaluation of HRQoL as a self-perceived physical and mental status over time is increasingly recognized as a pivotal outcome complementing traditional parameters, such as mortality^8^.

The current study aimed to determine HRQoL after COVID-19 hospitalization utilizing a longitudinal assessment in non-ICU and ICU COVID-19 patients. Instruments providing an overall summary of HRQoL and specific instruments focusing on depression, anxiety, stress, and fatigue were utilized.

## Methods

### Study design, patient population and data collection

This is a single-center longitudinal study conducted at the University Hospital of Würzburg, Germany. Potential patients were identified from a prospective, daily screening log between March and December 2020. Adult patients  ≥ 18 years of age were eligible for enrollment if they required inpatient treatment at the University Hospital of Würzburg with COVID-19. All COVID-19 patients admitted to the University Hospital of Würzburg during the respective time were evaluated for study inclusion. SARS-CoV-2 infection had to be confirmed with real-time reverse transcriptase polymerase chain reaction (RT-PCR) testing. None of the patients was vaccinated against SARS-CoV2 during acute illness, as vaccines were not available during the respective time. Baseline assessment was done on the day of hospital admission. Patients were categorized according to the need of ICU or non-ICU treatment, respectively.

Patient socio-demographics (including gender, age, body mass index -BMI, prior work status, and education), comorbidities, clinical characteristics (e.g. invasive mechanical ventilation, non-invasive mechanical ventilation, high-flow oxygen, tracheostomy, extracorporeal membrane oxygenation -ECMO), complications, and outcome were recorded at the time of inpatient COVID-19 treatment. All data were retrieved via patient data management systems (PDMS) (COPRA6 RM1.0, COPRA System GmbH, Berlin, Germany; Meona Produktion, Mesalvo Freiburg GmbH, Freiburg, Germany), as well as personal communication. Data were documented within a standardized electronic case report form (REDCap®, Vanderbilt University).

### Follow-up protocol and instruments

We evaluated patients 3 and 12 months after discharge from the University Hospital of Würzburg. At each visit, the patient was interviewed by telephone. The telephone interviews were scripted and performed by trained personnel. Questionnaires included the European Quality of Life 5 Dimensions 5 Level (EQ-5D-5L) as a general instrument of patient reported HRQoL. The five dimensions of the EQ-5D-5L are: mobility, self-care, usual activities, pain/discomfort, anxiety/depression. The EQ-5D-5L index value was calculated using the German value set. The patient health questionnaire-9 (PHQ-9) was used to measure depression. The PHQ-9 scores each of the 9 Diagnostic and Statistical Manual of Mental Disorders Version IV (DSM-IV) criteria as “0” (not at all) to “3” (nearly every day) as the respective module of the patient health questionnaire. The PHQ-9 has been found to have acceptable diagnostic properties for detecting major depressive disorder for cut-off scores between 8 and 11^9^. Based on this diagnostic window a cut-off value of ≥ 10 was chosen in the current study. The generalized anxiety disorder 7 (GAD-7) screened for the presence of a generalized anxiety disorder utilizing 7 items scored from “0” (not at all) to “3” (nearly every day). A score of 10 or greater on the GAD-7 represents a reasonable cut point for identifying cases of GAD^10^. Individual’s level of fatigue was assessed with the FACIT fatigue scale containing 13-items. The level of fatigue is measured on a scale from “4” (not at all fatigued) to “0” (very much fatigued). Scores < 30 indicate severe fatigue; higher scores indicate less fatigue. Stress assessment was done with the perceived stress scale (PSS-10). The PSS-10 contains 10 questions scored from “0” (never) to “4” (very often), whereas scores for questions 4, 5, 7, 8 are reversed. Scores ranging from 0 to 13 would be considered low stress, scores ranging from 14 to 26 would be considered moderate stress, scores ranging from 27 to 40 would be considered high perceived stress, respectively.

Moreover, posttraumatic stress (PTS) was investigated with the posttraumatic symptom scale 10 (PTSS-10). Response alternatives were ranked from “0” (never) to “3” (often). The PTSS-10 total score (score range = 0–30) constitutes the summation of the ratings and was interpreted according to the following levels of PTS-symptoms: 0 to 11 (mild to moderate) and 12 to 30 (moderate to severe range). A score of 18 or more represented a “case” and a score of 12 or above regarded as the need for psychological help. FACIT, PSS-10 and PTSS-10 were only collected at 12 months. All questionnaires were available as validated German language versions^11–15^.

### Functional status

Items on functional status included the ability of returning to work, reasons not to return to work, part in life as compared to prior to COVID-19 hospitalization (and housing status) at 3- and 12-months after hospital discharge. Clinical frailty was assessed as a baseline characteristic, as well as during the 3- and 12-months follow-ups.

### Statistical analysis

Continuous variables are presented as median and interquartile range (IQR), categorical variables as percentages. Two-tailed matched pairs Wilcoxon signed rank test was used for the analysis of ordinal data, two-tailed Mann–Whitney *U* test for unpaired ordinal data, Pearson’s Chi-squared test for categorical variables or McNemar testing to compare paired proportions, respectively. Wilcoxon signed-rank as a more powerful test was preferred over the sign test. Spearman correlation was utilized for correlation analysis. A p-value of ≤ 0.05 was considered statistically significant. Statistical calculations were performed using GraphPad Prism 9 (San Diego, California USA, 2020).

### Ethics

The study was performed in accordance with the Declaration of Helsinki. The Ethics Committee of the Medical Faculty of the Julius-Maximilians-University of Würzburg approved the study protocol (89/20-sc). Written informed consent was obtained from each patient or its legal representative prior to study inclusion.

## Results

**Figure 1:**
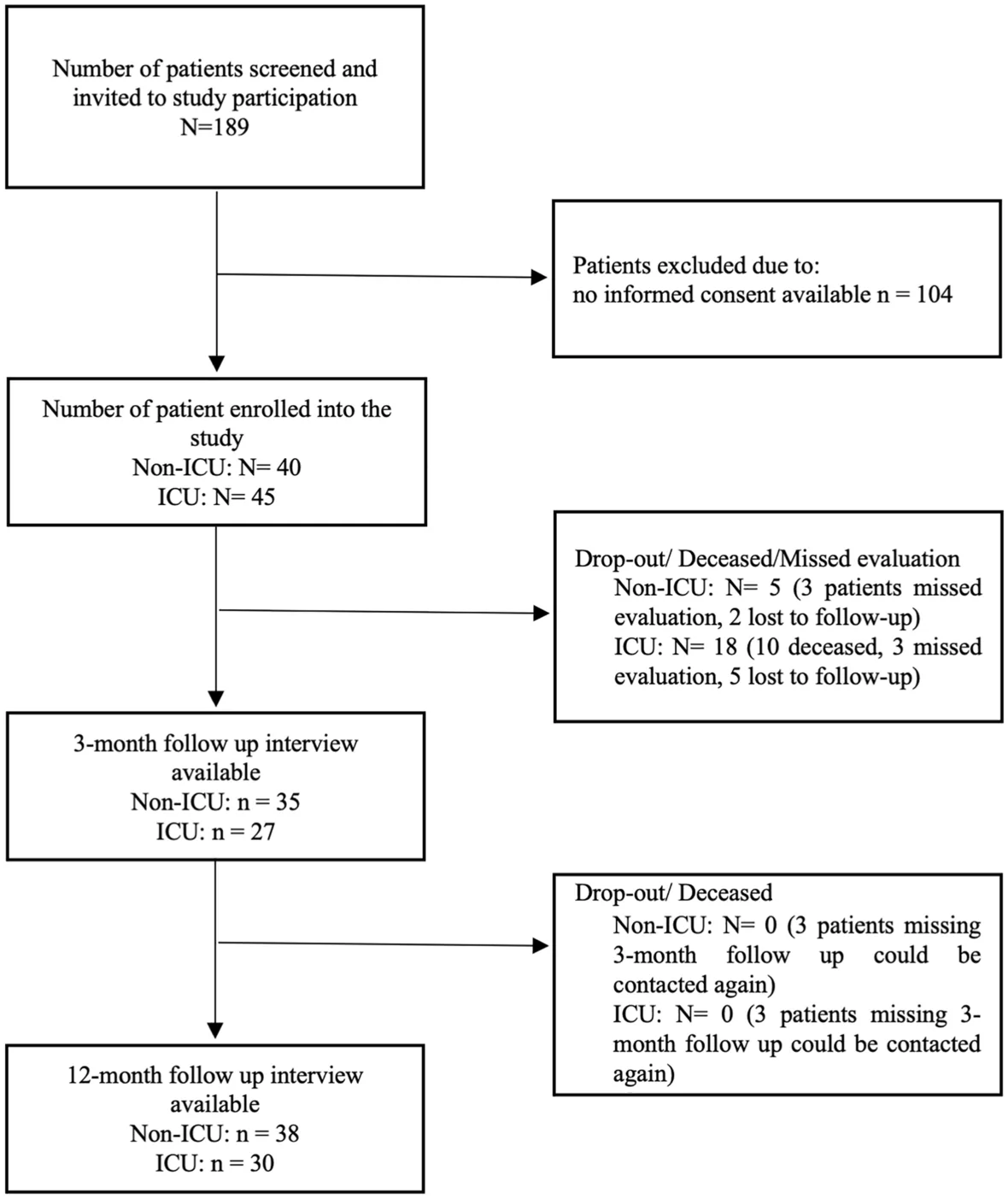
Screening and follow-up. Patients with COVID-19 treated at the University hospital of Würzburg were prospectively screened for study inclusion upon admission. After written consent, baseline characteristics were obtained and follow-up telephone interviews were conducted after 3- and 12-months, respectively.

Consent was not obtainable in 104 out of 189 eligible patients. A total of 85 patients were included in the study. 45 patients had critical COVID-19 with the need for ICU treatment. 3-month follow-up was available in 35 and 12-month follow-up in 38 non-ICU patients. A total of 27 ICU patients were evaluated during the 3-month follow-up, 30 ICU patients were evaluated during the 12-month follow-up (Fig. 1). COVID-19 was the primary diagnosis for hospital admission in 72 (85%) of the patients.

### Patient demographics

Patient demographics and comorbidities are depicted in Table 1. Median age was 63 (54–73) in non-ICU patients and 61 (52–68) in ICU patients, respectively. BMI was significantly higher in ICU (29 kg/m^2^) compared to non-ICU patients (26 kg/m^2^) (p = 0.0007). Age and BMI only had weak correlations to the questionnaire scores (Fig. S3). Arterial hypertension was the most common comorbidity. Most patients had 5 or 6-year secondary school education. Prior to hospitalization 74% non-ICU and 62% ICU patients categorized in a clinical frailty scale of 1–3 (very fit – managing well), with no significant differences between the two groups (p = 0.0998).

**Table 1:** Patient demographics and comorbidities at baseline.

Characteristic	Non-ICU (N = 40)	ICU (N = 45)
Age (years)	63 (54–73)	61 (52–68)
Male (%)	70	64
BMI (kg/m^2^)	26 (24–29) (N = 39)	29 (26–35) (N = 42)
Education n (%)	(N = 34)	(N = 30)
None	2 (6)	2 (7)
Five-year secondary school	10 (29)	10 (33)
Six-year secondary school	7 (21)	10 (33)
Grammar school	6 (18)	3 (10)
University/college degree	9 (26)	5 (17)
Cardiovascular risk factors n (%)		
Arterial hypertension	19 (48)	26 (58)
Coronary artery disease	4 (10)	5 (11)
Heart failure	4 (10)	0
Pulmonary risk factors n (%)		
Chronic obstructive pulmonary disease	5 (13)	2 (4)
Bronchial asthma	7 (18)	5 (11)
Obstructive sleep apnea	0	2 (4)
Neurologic disorder n (%)		
Apoplex cerebri	0	0
Parkinson’s disease	2 (5)	2 (4)
Multiple sclerosis	0	0
Psychatric disorder n (%)		
Dementia	2 (5)	0
Depression	3 (8)	2 (4)
Psychosis	1 (3)	0
Anxiety disorder	0	1 (2)
Clinical frailty scale n (%)	(N = 35)	(N = 34)
Very fit (1)	4 (11)	2 (6)
Well (2)	15 (43)	10 (29)
Managing well (3)	7 (20)	9 (26)
Vulnerable (4)	4 (11)	6 (18)
Mildly frail (5)	4 (11)	1 (3)
Moderately frail (6)	0	2 (6)
Severely frail (7)	1 (3)	3 (9)
Very severely frail (8)	0	1 (3)
Terminally ill (9)	0	0

Values are depicted as median plus interquartile range, or percentage of patients as indicated.

*ICU* intensive care unit, *BMI* body mass index.

### Clinical characteristics and outcome

Nearly half of the patients required high-flow oxygen treatment during non-ICU care, while 89% of the ICU patients were mechanically ventilated. Moreover, ECMO support was necessary in 44% of the ICU patients and renal replacement therapy in 36%. Pulmonary co-infections were found in 5% of non-ICU (two patients) and 38% of the ICU patients. Bloodstream co-infections were present in 8% of non-ICU (three patients) and 38% of the ICU patients. All non-ICU patients survived to hospital discharge or transfer to another hospital/skilled care facility, respectively. Mortality of ICU treatment was 22% (10 patients). There were no additional deaths in either group during the 12-month follow-up period. (Table S1).

### European quality of life 5 dimensions 5 level (EQ5D-5L)

The mean EQ5D-5L-index as an overall measure of the HRQoL differed between non-ICU (0.78 ± 0.33 and 0.84 ± 0.23) and ICU (0.71 ± 0.27; 0.74 ± 0.2) patients after 3- and 12-months with a significant difference after 12-months (p = 0.0511 and p = 0.0013, respectively) (Fig. S1 and Table 2). Mobility significantly differed between ICU and non-ICU patients after 12-months (p = 0.0155). Mobility was reported as no problem by one-third of ICU patients, whereas slight or moderate problems were present in a total of 40%, severe problems in 23%, respectively. However, the percentage of ICU patients with severe problems or unable to walk about decreased between the follow-ups. Usual activities were also significantly impaired in ICU compared to non-ICU patients at 3- (p = 0.0334) and 12-months (p = 0.0021), respectively. In non-ICU patients 58% were able to resume their usual activities after 12-months, one-third still suffered from slight or moderate problems. More than half of ICU patients had slight or moderate problems after 12-months, 20% had severe problems or were unable to perform their usual activities.

**Table 2:** Functional status as well as questionnaire results for general HRQoL.

Parameter	Non-ICU		ICU		p-value			
	3-month follow-up (N = 35)	12-month follow-up (N = 38)	3-month follow-up (N = 27)	12-month follow-up (N = 30)	Non-ICU 3-month vs. 12-month follow-up	ICU 3-month vs. 12-month follow-up	ICU vs. Non-ICU 3-month follow-up	ICU vs. Non-ICU 12-month follow-up
Living situation n (%)					> 0.999	0.2188	0.3328	05004
At home independent	29 (83)	33 (87)	19 (70)	24 (80)				
At home dependent	4 (11)	3 (8)	6 (22)	5 (17)				
Nursing home	2 (6)	2 (5)	2 (7)	1 (0.3)				
Change of living situation n (%)		6 (16)		6 (20)				0.7501
Daily activities n (%)	(N = 34)				0.7402	**0.0151**	**0.0033**	0.0942
Unchanged to pre-COVID-19	18 (53)	20 (53)	4 (15)	10 (33)				
Limited	10 (29)	14 (37)	13 (48)	15 (50)				
Severely limited	6 (18)	4 (12)	10 (37)	5 (17)				
Returned to work n (%)	16 (46)	18 (47)	10 (37)	11 (37)	> 0.999	0.3447	0.60599	0.3265
Reason if not	(N = 18)	(N = 19)	(N = 17)	(N = 19)				
Retired	16 (88)	17 (89)	10 (59)	11 (58)				
Unemployed	1 (6)	0	0	1 (5)				
Disability	1 (6)	2 (11)	7 (41)	7 (37)				
Clinical frailty scale n (%)				(N = 29)	0.2125	**0.0339**	**0.0014**	**0.0553**
Very fit (1)	6 (17)	5 (13)	0	2 (7)				
Well (2)	9 (26)	10 (26)	2 (7)	4 (14)				
Managing well (3)	8 (23)	10 (26)	9 (33)	6 (21)				
Vulnerable (4)	8 (23)	7 (18)	5 (19)	11 (38)				
Mildly frail (5)	2 (6)	1 (3)	5 (19)	2 (7)				
Moderately frail (6)	1 (3)	3 (8)	4 (15)	2 (7)				
Severely frail (7)	0	1 (3)	1 (4)	1 (3)				
Very severely frail (8)	1 (3)	1 (3)	1 (4)	1 (3)				
Terminally ill (9)	0	0	0	0				
EQ-5D-5L (score)								
Mobility	1 (1–3)	1 (1–2)	2 (1–3)	2 (1–3)	0.1712	> 0.999	0.3021	**0.0155**
Self-care	1 (1–1)	1 (1–1)	1 (1–2)	1 (1–2)	0.1250	0.8281	0.222	0.1136
Usual activities	1 (1–2)	1 (1–2)	2 (1–3)	2 (2–3)	0.7812	> 0.999	**0.0334**	**0.0021**
Pain/discomfort	2 (1–3)	1 (1–2)	2 (2–3)	2 (1–3)	0.0774	0.7964	0.4445	0.061
Anxiety/depression	1 (1–3)	1 (1–2)	2 (1–3)	2 (1–2)	0.4447	> 0.999	0.4671	0.2664
EQ VAS score	70 (60–84)	70 (52–84)	68 (50–80)	65 (50–76)	0.3357	0.9937	0.4132	0.1164
EQ-5D-5L-index [mean ± SD]	0.78 ± 0.33	0.84 ± 0.23	0.71 ± 0.27	0.74 ± 0.2	0.2965	0.7906	0.0511	**0.0013**
PHQ-9 (score)	(N = 34)							
Little interest or pleasure in doing things?	0 (0–1)	0 (0–1)	0 (0–1)	0 (0–1)				
Feeling down, depressed, or hopeless?	0 (0–1)	0 (0–1)	0 (0–1)	0 (0–1)				
Trouble falling or staying asleep, or sleeping too much?	1 (0–2)	1 (0–2)	1 (0–3)	1 (0–2)				
Tiredness, feeling of missing energy?	1 (1–2.5)	1 (0–2)	1 (0–3)	1 (0–2)				
Poor appetite or overeating?	0 (0–0.5)	0 (0–0)	0 (0–1)	0 (0–0)				
Feeling bad about yourself — or that you are a failure or have let yourself or your family down?	0 (0–0)	0 (0–0)	0 (0–0)	0 (0–0)				
Trouble concentrating on things?	0 (0–1)	0 (0–1)	0 (0–0.5)	1 (0–1)				
Moving or speaking so slowly that other people could have noticed? Or so fidgety or restless that you have been moving a lot more than usual?	0 (0–0)	0 (0–0)	0 (0–1)	1 (0–0.8)				
Thoughts that you would be better off dead, or thoughts of hurting yourself in some way?	0 (0–0)	1 (1–2)	0 (0–0)	2 (1–2)				
Total	4 (2–7.5)	3.5 (1–6)	5 (1.5–9.5)	5 (1.3–7.8)	0.1772	0.7002	0.7156	**0.0003**
Score ≥ 10 n (%)	6 (17)	7 (18)	7(26)	6 (20)			0.4329	0.5944
GAD-7								
Feeling nervous, anxious, or on edge	0 (0–1)	0 (0–1)	0 (0–1)	0.5 (0–1)				
Not being able to stop or control worrying	0 (0–1)	0 (0–0)	0 (0–0)	0 (0–1)				
Worrying too much about different things	0 (0–1)	0 (0–1)	0 (0–1)	0 (0–1)				
Trouble relaxing	0 (0–1)	0 (0–1)	0 (0–1)	0 (0–1)				
Being so restless that it's hard to sit still	0 (0–1)	0 (0–0)	0 (0–0.5)	0 (0–0)				
Becoming easily annoyed or irritable	0 (0–1)	0 (0–1)	0 (0–0)	0 (0–1)				
Feeling afraid as if something awful might happen	0 (0–0)	0 (0–0)	0 (0–0)	0 (0–1)				
Total	3 (1–6.5)	2 (0–4)	2 (0–4.5)	2.5 (0–5)	0.3544	0.6436	0.7236	0.6930
Score ≥ 10 n (%)	1 (3)	0	4 (15)	4 (13)			0.0864	**0.0203**

EQ-5D-5L (European Quality of Life 5 Dimensions 5 Level Version), PHQ-9 (Patient Health Questionnaire-9) and GAD-7 (Generalized Anxiety Disorder 7) Each of the five dimensions comprising the EQ-5D is divided into five levels of perceived problems (1: indicating no problem, 2: indicating slight problems, 3: indicating moderate problems, 4: indicating severe problems, 5: indicating unable to/extreme problems). EQ-5D-5L VAS is scaled from 0 (the worst health you can imagine) to 100 (the best health you can imagine). PHQ-9 and GAD-7 are a self-report measures, whereas respondents are asked to rate each of the items on a scale of 0 to 3 based on how much a symptom has bothered them over the last 2 weeks (0: not at all, 1: several days, 2: more than half the days, 3: nearly every day). Scores for each item are added up to get a total. A PHQ-9 score ≥ 10 was utilized for detecting major depressive disorder. A GAD-7 score ≥ 10 represents a reasonable cut point for identifying cases of GAD. Results are median plus interquartile range or percentage of patients as indicated. EQ-5D-5L-index values calculated using the German value set are mean ± SD.

Significant values are in bold.

**Figure 2:**
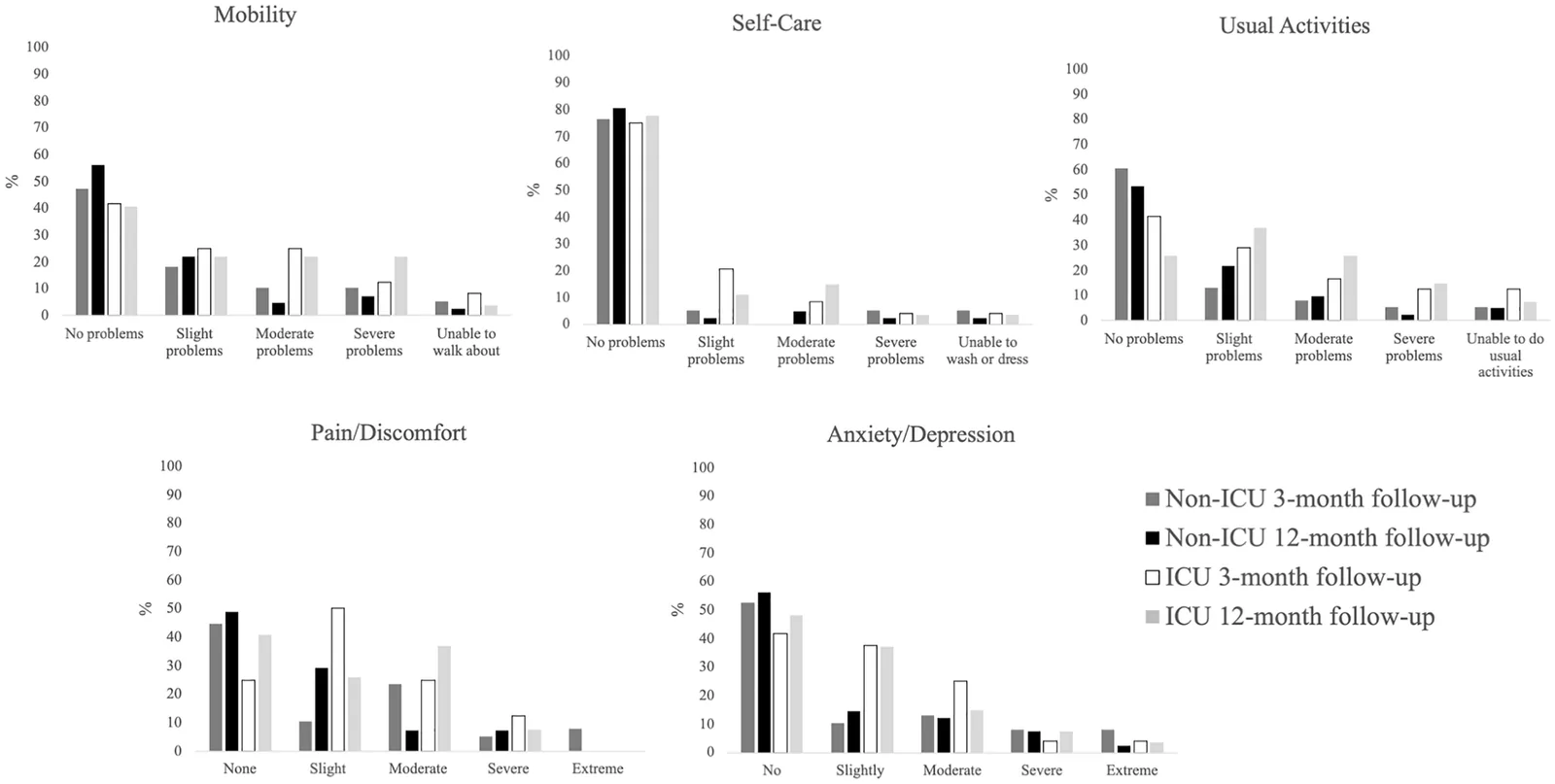
EQ5D-5L dimensions. Each of the five dimensions of the EQ5D-5L displayed as the percentage of patients within the respective category at the 3- and 12-month follow-ups. More than half of ICU patients were limited in their mobility with severe problems in 23% vs. 7% in non-ICU patients. In non-ICU patients 56% had no mobility problems vs. 41% in ICU patients. Moderate or slight problems in self-care were more common in ICU patients. However, in both ICU and non-ICU patients around 80% reported no problems after 12 months. Nevertheless, 15% of ICU patients had severe problems to perform their usual activities compared to 2% in non-ICU patients. Only 25% of the ICU patients reported no problems, compared to 53% in non-ICU patients after 12-months. Extreme pain was not observed in any group, while severe pain was present in 7% of both ICU and non-ICU patients. However, moderate pain remained in 37% of ICU patients compared to only 7% in non-ICU patients. Anxiety/depression was reported in similar percentages in both groups along all categories, with 10 to 11% having severe or extreme problems in total.

Self-care, pain/discomfort, anxiety/depression did not significantly differ between non-ICU and ICU patients, as well as between the 3-months and 12-months follow-up. No problems in self-care were present in most patients in both groups, whereas a higher percentage of ICU patients suffered from slight or moderate pain/discomfort or anxiety/depression. Severe or extreme problems were only present in a small percentage in both groups (Fig. 2).

### Functional status

Only two non-ICU and one ICU patient resided in a nursing home after 12 months. A significantly higher percentage of ICU patients remained limited in their daily activities after 3-months (p = 0.0033). However, recuperation happened between 3 and 12-months follow-up with five (17%) ICU patients not being severely limited anymore, and 6 (18%) more returning to their usual daily activities. Although clinical frailty was not significantly different at baseline (p = 0.0998), in non-ICU patients 10.5% of the patients fell into the categories moderately limited or worse after 12 months as a significant difference compared to baseline (p = 0.047). This was not the case in ICU patients (p = 0.0749), whereas clinical frailty significantly improved between the 3- and 12-month follow-up (p = 0.0339). Nevertheless, a high percentage of ICU patients remained vulnerable after 12-months with 38% still vulnerable. Only 6 percent were severely or very severely frail. Overall frailty in non-ICU patients did not change comparing 3- and 12-months follow-ups, with a total of 36% being very fit or well and another 26% managing well after 12-months. Only 37% of ICU patients and half of the non-ICU patients returned to work. Reason for not returning to work was retirement in 89% non-ICU and 58% ICU patients, whereas 11% of non-ICU and 37% of ICU patients were unable to work due to disability. Of non-ICU 87% and 80% of ICU patients were independently living at home after 12 months (Table 2).

### Depression, anxiety, fatigue and stress

None of the non-ICU patients had a GAD-7 score ≥ 10 at 12-month follow-up. Four ICU patients suffered from GAD at 3- and 12-months follow-up, which was significantly different compared to the non-ICU group (p = 0.0203) after 12-months. Results of the GAD-7 for anxiety did not significantly differ between non-ICU and ICU patients, as well as between the 3- and 12-months follow-up. PHQ-9 for depression significantly differed between ICU and non-ICU patients. However, considering a cutoff value of ≥ 10 for the PHQ-9, 26% of ICU patients had a depression after 3-months, which decreased to 20% after 12-months. This was not significantly different to non-ICU patients with percentages of 17% and 18%, respectively (Table 2).

FACIT fatigue scale was only assessed at the 12-months follow-up and did not result in significant differences between non-ICU and ICU patients, as 30% of the ICU patients and 21% of non-ICU patients fulfilled the diagnostic criterion for fatigue (Table 3).

**Table 3:** Questionnaire results testing for perceived stress, fatigue, and posttraumatic stress symptoms at the 12-month follow-up.

Parameter	Non-ICU	ICU	p-value
	12-month follow-up		
PSS-10 (score)	(N = 38)	(N = 30)	
In the last month, how often have you been upset because of something that happened unexpectedly?	2 (1–2)	2 (1–3)	
In the last month, how often have you felt that you were unable to control the important things in your life?	1 (1–2)	1 (1–3)	
In the last month, how often have you felt nervous and stressed?	2 (2–3)	3 (2–3)	
In the last month, how often have you felt confident about your ability to handle your personal problems?	5 (4–5)	4.5 (3–5)	
In the last month, how often have you felt that things were going your way?	4 (4–5)	4 (3–4)	
In the last month, how often have you found that you could not cope with all the things that you had to do?	2 (1–3)	3 (1–3)	
In the last month, how often have you been able to control irritations in your life?	4 (3–5)	3 (3–4)	
In the last month, how often have you felt that you were on top of things?	5 (4–5)	3 (3–5)	
In the last month, how often have you been angered because of things that happened that were outside of your control?	3 (2–3)	3 (-3)	
In the last month, how often have you felt difficulties were piling up so high that you could not overcome them?	1 (1–2)	2 (1–2)	
Total	19 (14–24)	20 (17–29.75)	0.0547
Score ≥ 27 n (%)	7 (18)	9 (30)	0.2637
Score ≤ 13 n (%)	9 (24)	1 (3)	**0.0186**
FACIT-F (score)	(N = 38)	(N = 30)	
I feel fatigued	1 (0–2)	1 (0–3)	
I feel weak all over	1 (0–2)	1 (0–3)	
I feel listless (“washed out”)	1 (0–2)	1 (0–2)	
I feel tired	0 (0–2)	2 (1–3)	
I have trouble starting things because I am tired	1 (0–2)	0.5 (0–2)	
I have trouble finishing things because I am tired	0 (0–1)	0 (0–2)	
I have energy	2 (2–3)	2 (1–3)	
I am able to do my usual activities	4 (2–4)	3 (1–4)	
I need to sleep during the day	1 (0–3)	2 (0–3)	
I am too tired to eat	0 (0–0)	0 (0–0)	
I need help doing my usual activities	0 (0–0)	0 (0–2)	
I am frustrated by being too tired to do the things I want to do	0 (0–1)	0 (0–1)	
I have to limit my social activity because I am tired	0 (0–1)	0 (0–1)	
Total	42 (33–50)	36 (23.5–47)	0.3268
Score ≤ 30 n (%)	8 (21)	9 (30)	0.3975
PTSS-10 (score)	(N = 38)	(N = 30)	
Sleep problems	1 (0–2)	2 (0–3)	
Nightmares about the events	0 (0–0)	0 (0–1)	
I feel dejected/downtrodden	0 (0–1)	1 (0–2)	
Jumpiness, I am easily frightened by sudden sounds I hear or sudden movements I see	0 (0–1)	0 (0–1)	
The need to withdraw from others	0 (0–1)	0 (0–1)	
Irritability	0 (0–1)	0.5 (0–2)	
Frequent mood swings	0 (0–2)	1 (0–2)	
Bad conscience, blame me, have guilt feelings	0 (0–0)	0 (0–1)	
Fear of places and situations, which remind me of the events	0 (0–0)	0 (0–0)	
Muscular tension	0 (0–1)	1 (0–2)	
Total	5 (0–10)	6.5 (4–11)	0.1471
Score ≥ 12 n (%)	6 (16)	7 (23)	0.4322
Score ≥ 18 n (%)	2 (5)	3 (10)	0.4574

PSS-10 (Perceived Stress Scale-10) respondents are asked to rate each of the items on a scale of 0 to 4 based on how much a symptom has bothered them during the last month (0: never, 1: almost never, 2: sometimes 3: fairly often 4: very often). Scores for questions 4, 5, 7, and 8 are inverted. On these 4 questions, the scores are changed like this: 0 = 4, 1 = 3, 2 = 2, 3 = 1, 4 = 0. Scores are subsequently added up to get a total. Scores ranging from 0 to 13 would be low stress, scores ranging from 14 to 26 would be considered moderate stress and scores ranging from 27 to 40 would be considered high-perceived stress, respectively. FACIT-F (Functional Assessment of Chronic Illness Therapy – Fatigue) respondents are asked to rate each of the items on a scale of 0 to 4 based on the past 7 days. Scores on items 7 and 8 are reversed like this: 0 = 4, 1 = 3, 2 = 2, 3 = 1, 4 = 0. Scores are subsequently added up to a total. Scores < 30 indicate severe fatigue. PTSS-10 (Posttraumatic Symptom Scale-10) respondents are asked to rate each of the items on a scale of 0 to 3 (0: never, 1: rarely, 2: sometimes 3: often). PTSS-10 scores are subsequently added up to get a total.scores interpreted according to the following levels: 0 to 11 (mild to moderate) and 12 to 30 (moderate to severe). A score ≥ 18 represented a “case” and a score of ≥ 12 indicates the need for psychological help. Results are median plus interquartile range or percentage of patients as indicated.

Significant values are in bold.

Stress levels remained high in both non-ICU and ICU patients. Only 24% of non-ICU and 3% of ICU patients (p = 0.0186) had low perceived stress on the PSS. Relative risk of ICU patients not having a PSS-score ≤ 13 was 7.1 (CI 95% 1.3–42.2). High perceived stress was present in 30% of the ICU patients and 18% of non-ICU patients at 12-months.

Posttraumatic symptoms were not present in as many patients, with only 5% of non-ICU and 10% of ICU patients having a PTSS-10 ≥ 18, indicating a posttraumatic stress disorder. There was no significant difference between the patient groups (Table 3). The 12-months follow-up results for depression, anxiety, fatigue, and stress are visualized in Fig. S2.

## Discussion

Severe COVID-19 is associated with high in-hospital mortality and long-lasting limitations. In the current study HRQoL was reduced in COVID-19 ICU patients 3- and 12-months post COVID-19 hospitalization, with significantly less improvement at 12-months compared to non-ICU patients. Mobility and usual activities were most severely impeded. Approximately one-fifth of both non-ICU and ICU patients suffered from depression. Severe fatigue and stress were also present in a high number of patients, whereas GAD and PTS were only observed in a small fraction.

The utilized EQ5D-5L is widely applicable as an instrument of general HRQoL and can be used in quality-adjusted life year (QALY) calculations. Mean EQ5D-5L indexes in non-ICU patients were similar to what has been described in the German population aged 55- 64 years (0.87^16^) or > 65 years (0.84–0.88^16,17^), respectively. Although our cohort might not be directly comparable to the general population of the same age, the percentage of non-ICU patients reporting no problems in the five different EQ-5D-5L dimensions was lower. In the general population up to two-thirds report no problems across all dimensions^18^. This indicates that in non-ICU cases, although recovery to a decent HRQoL was observed, long-term sequelae are common. In ICU patients all dimensions were strikingly limited. Only one-third reported no problems with mobility, pain/discomfort, and anxiety/depression. Less than one fourth were able to do all their usual activities. In conjunction with low median VAS scores and low EQ-5D-5L index scores, our data indicate that COVID-19 ICU patients still suffer from a significantly lower HRQoL after 12-months. EQ-5D-5L index scores were like what has been reported in mild heart failure (NYHA class I/II)^19^ or survivors of other causes of acute respiratory distress syndrome (ARDS). Results in non-COVID-19 ARDS survivors were like our data with EQ-5D-VAS scores of 69 and index scores of 0.75^20^. This is further corroborated by a systematic review of 4408 patients identifying ICU treatment as a risk factor of low HRQoL^21^. A meta-analysis showed that ARDS, prolonged mechanical ventilation, and sepsis lead to significant impairments in long-term QoL, whereas survivors of cardiac arrest, severe pancreatitis, esophagectomy, and acute kidney injury had a comparable or even better QoL than age- and gender-matched populations. It is important to notice, that reduced HRQoL is not equal to an unacceptable outcome. In a study of 1453 post-ICU patients, 95% self-reported their outcome acceptable irrespective of a reduced HRQoL with an EQ-5D-index of 0.81. Those reporting an unacceptable outcome had an EQ-5D-index of 0.57 while EQ-5D-index cut-off values could not be defined. Besides a low EQ-5D-index, symptoms of anxiety and depression had the strongest association with self-reported unacceptable outcome one year after ICU discharge^22^. Although these data originate from non-COVID-19 patients, it may be reasonable to assume a similar perception in our study cohort. Nevertheless, while physical improvement may take years, mental and emotional aspects are often stagnant or decline even further^23^.

Long-term mental health problems are recognized as COVID-19 consequences impeding full-recovery^24^. Of our non-ICU patients 18% and 20% of our ICU patients tested at risk for a major depressive disorder after 12-months, whereas it needs to be considered that the PHQ-9 cutoff-value of 10 may result in many false negatives in hospital settings and more false positives in primary care^9^. Nevertheless, a large community-based study in Great Britain found a similar overall prevalence of anxiety/depression (26.4%) and only a small positive association to SARS-CoV-2 infection^25^. Depression is highly interconnected to fatigue^26^, which was found in a similar percentage of patients. Fatigue may be debilitating. and although it improves over time, a meta-analysis showed that population references scores are seldomly reached^27^. In our cohort, a lower fraction of ICU patients had fatigue compared to what had been previously reported in prolonged critical illness with critical illness polyneuropathy/myopathy^28^. Moreover, the percentage of ICU patients with a suspected anxiety disorder was lower than what has been reported at one month follow-up^24^ or in the COVID symptom study^25^. Anxiety is often associated with younger adults and females, whereas the median age in our study was older and two-thirds were male. Furthermore, as depression or anxiety symptoms increased in the general population during the pandemic due to disrupted work or social life, the overall assessment of depression, fatigue and anxiety as specific post-COVID-19 symptoms is complicated^29^ and cannot be reasoned from our data.

Another important aftermath of severe disease and hospitalization is perceived stress or posttraumatic stress disorder (PTSD). PTSD has been described in up to 20% of general ICU survivors, whereas it is less common in non-ICU patients^30,31^. Trauma of hospitalization is furthermore associated with greater risk of 30-day readmission^32^. In our cohort, 10% of COVID-19 ICU patients had suspected PTSD, while a total of 23% were at risk. This in line with results from the BRAIN-ICU trial, finding PTSD in 7% of patients after 3 and 12 months^33^. It is of note, that we found a comparably high rate of suspected PTSD in non-ICU patients. While symptoms of PTSD, i.e. evidenced by the presence of flashbacks or nightmares, jumpiness, or fear of reminiscent situations were altogether rarely observed, symptoms of perceived stress were three times as common in ICU patients. Perceived stress is characterized by an individual’s appraise of situations in their lives as excessively uncontrollable and the extent to which they feel overloaded. Not only a higher percentage of ICU patients had high levels of stress, although non-significant, but also a significantly lower percentage had low levels of stress compared to non-ICU treatment. Low levels of stress were rarely observed in ICU patients, indicating that nearly all ICU survivors experience moderate to high levels of stress one-year after discharge.

Limitations of our study include the single-center design, with a high percentage of ECMO patients as the University hospital of Würzburg is a regional ECMO/ARDS referral center. Nearly half of our ICU cohort required ECMO support, which had been associated with long-term limitations in daily life^34^. However, others have also shown an overall positive long‐term HRQoL outcome for ECMO survivors with only few pulmonary and mental limitations^35,36^. We also found a high level of bacterial co-infections in this severely ill cohort. Both factors could potentially limit external validity. Furthermore, we found a significantly higher baseline BMI in ICU compared to non-ICU patients. In prior studies increasing BMI was associated with a higher probability of low self-perceived HRQoL and self-reported problems in all five EQ5D-5L dimensions. Effects of BMI on HRQoL were strongest in patients with severe to morbid obesity (BMI ≥ 35) and primarily affected mobility and pain/discomfort^37^. Hence, we cannot exclude that the higher BMI in ICU patients to some extent explains the lower HRQoL. Nevertheless, we only found a negligible negative correlation between EQ5D-5L-Index and BMI. Nutrition problems in ICU patients are complex and it needs to be considered that critically ill patients mostly lose lean body mass during ICU treatment. Although weight is subsequently gained back, nearly all of this is fat mass and not functional lean body mass. A catabolic/hypermetabolic state can maintain for up to 2 years after ICU discharge^38,39^, emphasizing the importance of individual nutrition management post ICU.

Consent was not obtainable in 55% of all eligible patients. Reasons include refusal to participate, as well as language barrier or unavailability of a legal representative amongst others. This low response rate could have potentially resulted in a selection bias. Due to the prospective design with the need for informed consent we cannot exclude a selection consent bias^40^. All patients included in the study were admitted as emergency hospitalizations. As such patients were not known prior to admission and prior recording of EQ-5D-5L was not possible. Overall clinical frailty was not significantly different at baseline. However, a higher proportion of ICU patients was moderately, severely, or very severely frail, which could have affected HRQoL at baseline, 3 and 12 months. The different EQ5D-5L health states were converted into a single index using country specific value sets, in our case representing the societal preferences of the German population^41^. We did not collect data on pulmonary function or six-minute walking distance. Hence, we cannot not correlate our data on HRQoL with follow-ups of physical status. Functional recovery after COVID-19 ARDS defined either by alterations on pulmonary function tests, a standardized 6 min walk test or fibrosis-like pulmonary findings will be further investigated in the “Functional Recovery From Acute Respiratory Distress Syndrome (ARDS) Due to COVID-19: Influence of Socio-Economic Status” (RECOVIDS) study^42^. The questionnaires used are generic scales, not specifically designed for HRQoL post COVID-19. They do not comprehensively consider all factors affecting HRQoL during the pandemic. Socioeconomics, lockdown measures, but also how COVID-19 affected family relationships (e.g. living alone or not, having relatives/friends who died) might have confounded the results. Furthermore, the presence of fatigue may be underrated as the FACIT-F was mainly developed for the geriatric population^13^. All results are based on univariate analysis and adjustment for possible confounders was not performed. Hence, we cannot exclude the possibility of interactions of the variables or one of the variables confounding other variables being studied.

In summary, our data highlight the complexity of post-COVID-19 symptoms as well as the necessity to educate patients and primary care providers about monitoring mental well-being. Particularly ICU survivors had a reduced HRQoL, like what has been described in ARDS from other causes. Mental disorders were common with high levels of perceived stress, depression, and fatigue. PTSD or anxiety on the contrary were less observed. However, our data also suggest that most patients were able to live independently at home after 12-months.

## Supplementary Information

Supplementary Figure S1.

Supplementary Figure S2.

Supplementary Figure S3.

Supplementary Table S1.

## Data Availability

The datasets used and/or analysed during the current study are available from the corresponding author on reasonable request.
