# Correction: Prospective single-center study of health-related quality of life after COVID-19 in ICU and non-ICU patients

**DOI:** 10.1038/s41598-026-64398-8

**Published:** 2026-08-25

**Authors:** Johannes Herrmann, Kerstin Müller, Quirin Notz, Martha Hübsch, Kirsten Haas, Anna Schäfer, Julia Schmidt, Peter Heuschmann, Jens Maschmann, Matthias Frosch, Jürgen Deckert, Hermann Einsele, Georg Ertl, Stefan Frantz, Patrick Meybohm, Christopher Lotz

**Affiliations:** 1 Department of Anaesthesiology, Intensive Care, Emergency and Pain Medicine, University Hospital Würzburg, Julius-Maximilians-University Wuerzburg, Würzburg, Germany; 2 Institute for Clinical Epidemiology and Biometry, Julius-Maximilians-University Würzburg, Würzburg, Germany; 3 Clinical Trial Center, University Hospital Wuerzburg, Würzburg, Germany; 4 University Hospital Würzburg, Julius-Maximilians-University Würzburg, Würzburg, Germany; 5 University Hospital Würzburg, Julius-Maximilians-University Wuerzburg, Würzburg, Germany; 6 Department of Psychiatry, Psychosomatics and Psychotherapy, University Hospital Würzburg, Julius-Maximilians-University Würzburg, Würzburg, Germany; 7 Department of Internal Medicine II, University Hospital Würzburg, Julius-Maximilians-University Würzburg, Würzburg, Germany; 8 Comprehensive Heart Failure Center Würzburg (CHFC), University Hospital Würzburg, Julius-Maximilians-University Wuerzburg, Würzburg, Germany; 9 Department of Internal Medicine I, University Hospital Würzburg, Julius-Maximilians-University Würzburg, Würzburg, Germany; 10 Department of Anaesthesiology, Intensive Care, Emergency and Pain Medicine, University Hospital Würzburg, Oberduerrbacherstr. 6, 97080 Würzburg, Germany

Correction to: *Scientific Reports* 10.1038/s41598-023-33783-y, published online 26 April 2023

Since the publication of this work, Anna Schäfer has changed their name from Anna Horn.

As a result, the Author Contribution section now reads:

“J.H., K.M., K.H., P.H., P.M. and C.L. conceptualized the study. K.M., Q.N., M.E.H., A.S., J.S. performed telephone surveys. J.H., K.H., A.S., J.S. and C.L. analysed data and prepared figures. J.H., K.H., P.H., P.M. and C.L. wrote the manuscript. All authors reviewed the manuscript.”

The original HTML and PDF versions of this Article have been corrected.

