# Supplementary material for: Prospective single-center study of health-related quality of life after COVID-19 in ICU and non-ICU patients
Source: Sci Rep. 2023 Apr 26;13:6785. doi: 10.1038/s41598-023-33783-y (PMC10133285; doi:10.1038/s41598-023-33783-y)
Supplement: Supplementary file 1 — Supplementary Figure S1. [file 41598_2023_33783_MOESM1_ESM.pptx]

## Slide 1
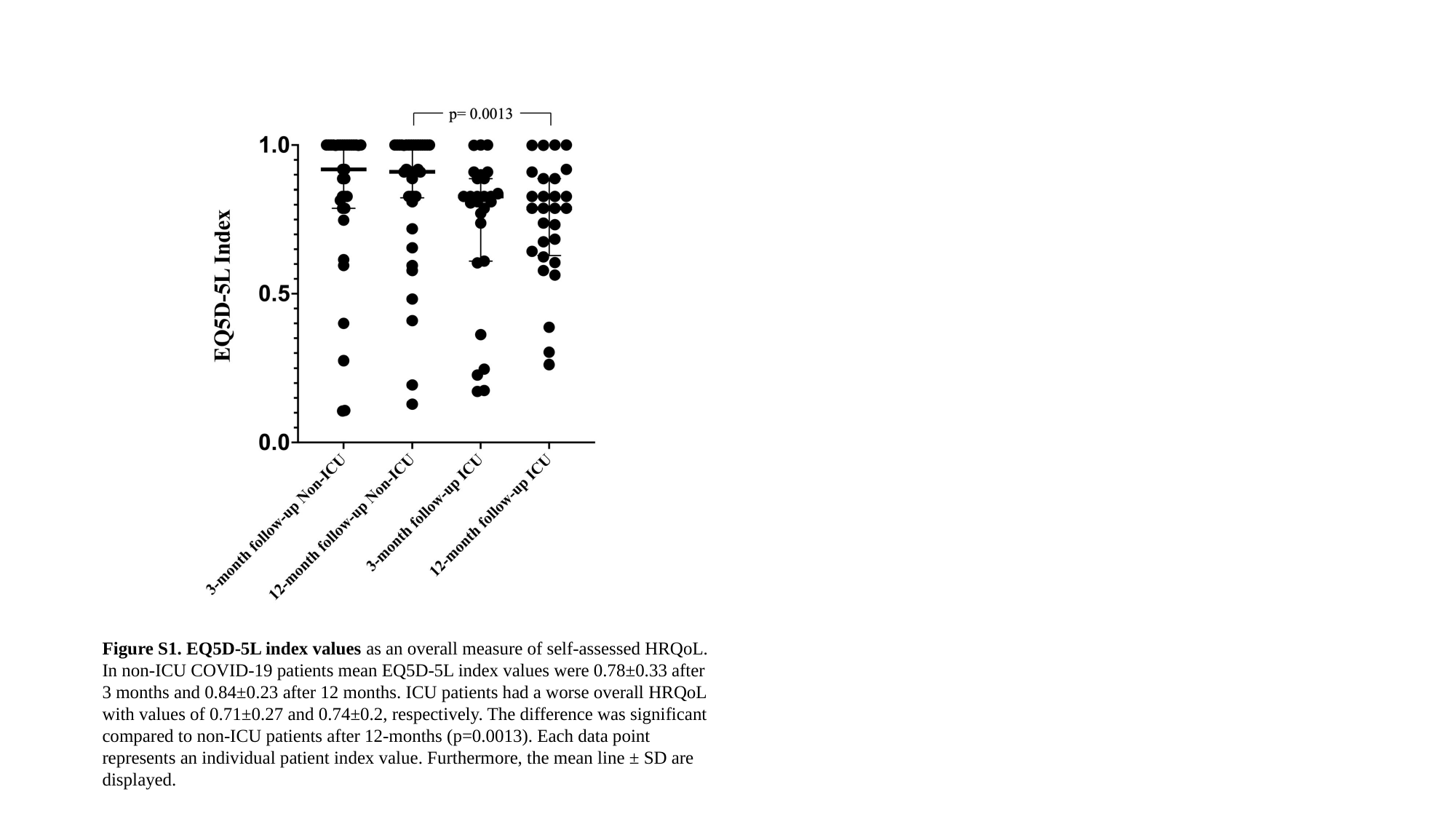

Figure S1. EQ5D-5L index values as an overall measure of self-assessed HRQoL.
In non-ICU COVID-19 patients mean EQ5D-5L index values were 0.78±0.33 after
3 months and 0.84±0.23 after 12 months. ICU patients had a worse overall HRQoL
with values of 0.71±0.27 and 0.74±0.2, respectively. The difference was significant
compared to non-ICU patients after 12-months (p=0.0013). Each data point
represents an individual patient index value. Furthermore, the mean line ± SD are
displayed.
