# Supplementary material for: Prospective single-center study of health-related quality of life after COVID-19 in ICU and non-ICU patients
Source: Sci Rep. 2023 Apr 26;13:6785. doi: 10.1038/s41598-023-33783-y (PMC10133285; doi:10.1038/s41598-023-33783-y)
Supplement: Supplementary file 2 — Supplementary Figure S2. [file 41598_2023_33783_MOESM2_ESM.pptx]

## Slide 1
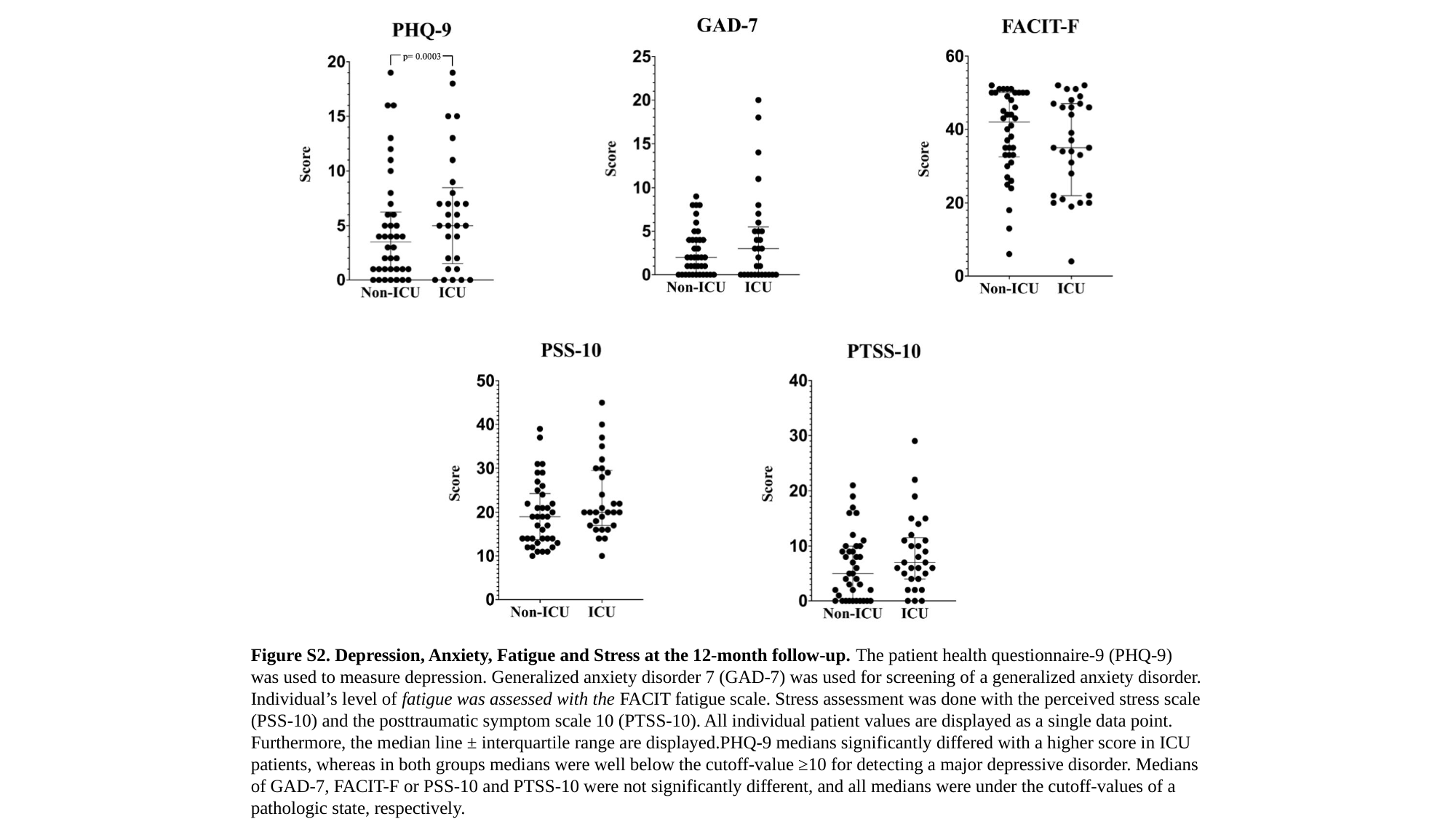

Figure S2. Depression, Anxiety, Fatigue and Stress at the 12-month follow-up. The patient health questionnaire-9 (PHQ-9) was used to measure depression. Generalized anxiety disorder 7 (GAD-7) was used for screening of a generalized anxiety disorder. Individual’s level of fatigue was assessed with the FACIT fatigue scale. Stress assessment was done with the perceived stress scale (PSS-10) and the posttraumatic symptom scale 10 (PTSS-10). All individual patient values are displayed as a single data point. Furthermore, the median line ± interquartile range are displayed.PHQ-9 medians significantly differed with a higher score in ICU patients, whereas in both groups medians were well below the cutoff-value ≥10 for detecting a major depressive disorder. Medians of GAD-7, FACIT-F or PSS-10 and PTSS-10 were not significantly different, and all medians were under the cutoff-values of a pathologic state, respectively.
