# Supplementary material for: Prospective single-center study of health-related quality of life after COVID-19 in ICU and non-ICU patients
Source: Sci Rep. 2023 Apr 26;13:6785. doi: 10.1038/s41598-023-33783-y (PMC10133285; doi:10.1038/s41598-023-33783-y)
Supplement: Supplementary file 3 — Supplementary Figure S3. [file 41598_2023_33783_MOESM3_ESM.pptx]

## Slide 1
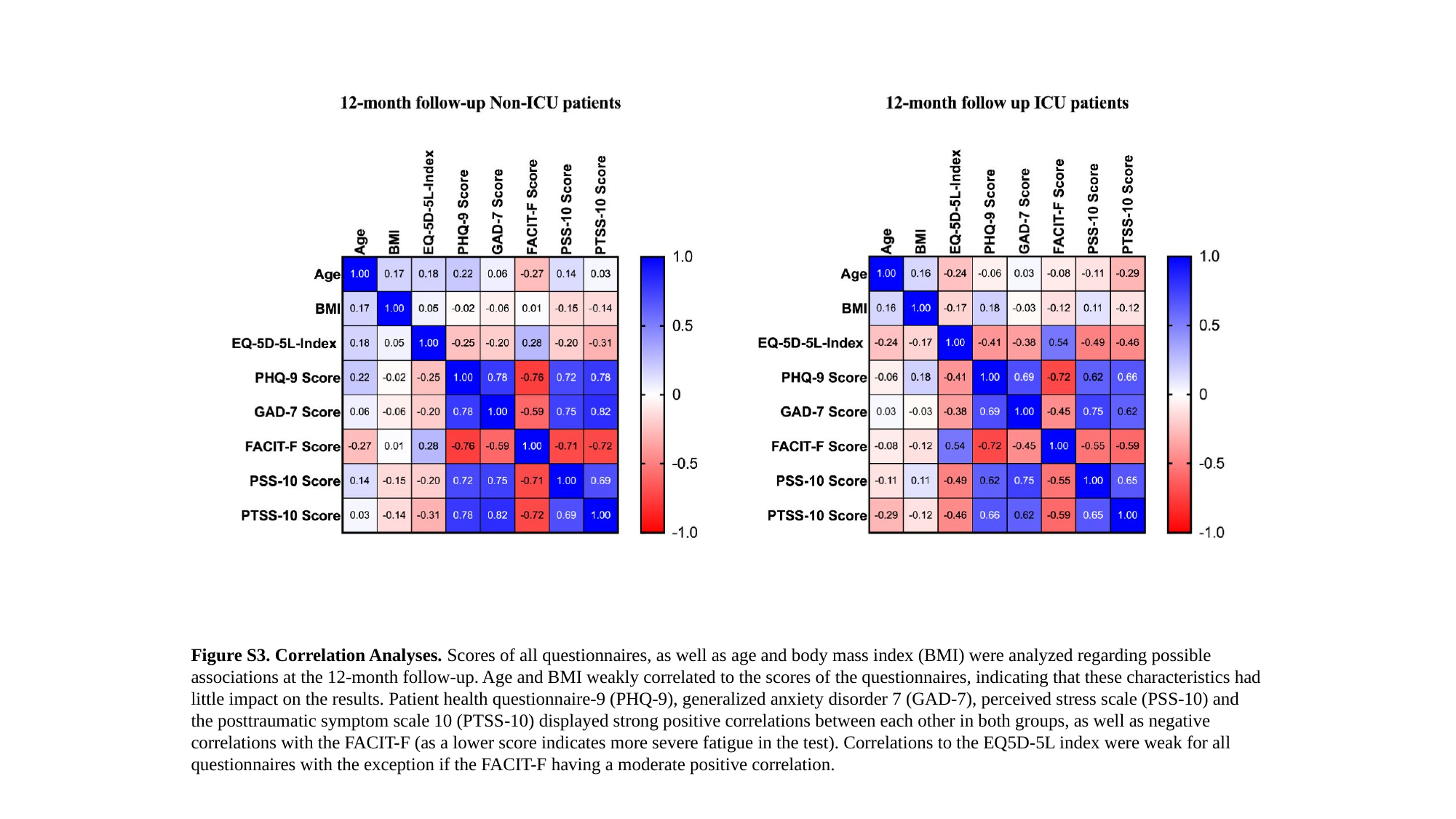

Figure S3. Correlation Analyses. Scores of all questionnaires, as well as age and body mass index (BMI) were analyzed regarding possible associations at the 12-month follow-up. Age and BMI weakly correlated to the scores of the questionnaires, indicating that these characteristics had little impact on the results. Patient health questionnaire-9 (PHQ-9), generalized anxiety disorder 7 (GAD-7), perceived stress scale (PSS-10) and the posttraumatic symptom scale 10 (PTSS-10) displayed strong positive correlations between each other in both groups, as well as negative correlations with the FACIT-F (as a lower score indicates more severe fatigue in the test). Correlations to the EQ5D-5L index were weak for all questionnaires with the exception if the FACIT-F having a moderate positive correlation.
