# Supplementary material for: Prospective single-center study of health-related quality of life after COVID-19 in ICU and non-ICU patients
Source: Sci Rep. 2023 Apr 26;13:6785. doi: 10.1038/s41598-023-33783-y (PMC10133285; doi:10.1038/s41598-023-33783-y)
Supplement: Supplementary file 4 — Supplementary Table S1. [file 41598_2023_33783_MOESM4_ESM.docx]

**Table S1.** Clinical Characteristics, Complications and Outcome. ECMO= extracorporeal membrane oxygenation

| Characteristic |  | Non-ICU (N=40) | ICU (N=45) |
| --- | --- | --- | --- |
| High-flow oxygen n (%) |  | 18 (45) | 2 (4) |
| Invasive mechanical ventilation - No. patients n (%) |  | 0 | 40 (89) |
| Non-invasive mechanical ventilation n (%) |  | 0 | 1 (2) |
| Tracheostomy n (%) |  | 0 | 19 (42) |
| ECMO n (%) |  | 0 | 20 (44) |
| Renal Replacement Therapy n (%) |  | 0 | 16 (36) |
| Pulmonary co-infection n (%) |  | 2 (5) | 17 (38) |
| Bloodstream infection n (%) |  | 3 (8) | 17 (38) |
| Thrombembolic event (venous) n (%) |  | 0 | 6 (13) |
| Pulmonary emboli n (%) |  | 0 | 4 (9) |
| Stroke n (%) |  | 0 | 2 (4) |
| Outcome n (%) | Hospital discharge (home) | 36 (90) | 15 (33) |
|  | Transfer to another hospital/skilled care facility | 4 (10) | 20 (44) |
|  | Deceased | 0 | 10 (22) |
